# Probability-based preservational variations within the early Cambrian Chengjiang biota (China)

**DOI:** 10.7717/peerj.13869

**Published:** 2022-08-23

**Authors:** Farid Saleh, Xiaoya Ma, Pauline Guenser, M. Gabriela Mángano, Luis A. Buatois, Jonathan B. Antcliffe

**Affiliations:** 1 MEC International Joint Laboratory for Palaeobiology and Palaeoenvironment, Yunnan University, Kunming, China; 2 Yunnan Key Laboratory for Palaeobiology, Yunnan University, Kunming, China; 3 Centre for Ecology and Conservation, University of Exeter, Penryn, United Kingdom; 4 University Bordeaux, CNRS, Bordeaux INP, EPOC, UMR5805, Pessac, France; 5 Department of Geological Sciences, University of Saskatchewan, Saskatoon, Canada; 6 Institute of Earth Sciences, Université de Lausanne, Lausanne, Switzerland

**Keywords:** Exceptional preservation, Preservation variation, Paleozoic, Cambrian, Decay, Taxonomic recognition

## Abstract

The Chengjiang biota (Yunnan Province, China) is a treasure trove of soft-bodied animal fossils from the earliest stages of the Cambrian explosion. The mechanisms contributing to its unique preservation, known as the Burgess Shale-type preservation, are well understood. However, little is known about the preservation differences between various animal groups within this biota. This study compares tissue-occurrence data of 11 major animal groups in the Chengjiang biota using a probabilistic methodology. The fossil-based data from this study is compared to previous decay experiments. This shows that all groups are not equally preserved with some higher taxa more likely to preserve soft tissues than others. These differences in fossil preservation between taxa can be explained by the interaction of biological and environmental characteristics. A bias also results from differential taxonomic recognition, as some taxa are easily recognized from even poorly preserved fragments while other specimens are difficult to assign to higher taxa even with exquisite preservation.

## Introduction

The early Cambrian Chengjiang biota (Epoch 2, Stage 3, Yunnan Province, China) records extraordinary evidence of early animal life on Earth. This fossil deposit shows exceptional preservation of soft-bodied animal macrofossils for both overall morphology and detailed anatomy, yielding a level of evolutionary and ecological data that is pivotal in our understanding of the very earliest history of animal life. Listed as a UNESCO World Heritage Site since 2012, the Chengjiang biota (https://whc.unesco.org/en/list/1388/) is particularly important because it is among the oldest animal fossil biotas (Zhu et al., 2019). Moreover, it captures an ecologically diverse assemblage, that colonized a deltaic environment, during the unfolding Cambrian Radiation (Ma et al., 2012; Ma et al., 2014; Tanaka et al., 2013; Cong et al., 2014; Liu et al., 2020a; Liu et al., 2020b; Liu et al., 2021; Yang et al., 2021; Saleh et al., 2022)—an event characterized by rapid rates of evolution and the emergence of most major animal groups (Daley et al., 2018). The mechanisms that led to the exceptional preservation of Chengjiang fossils are well studied (Gabbott et al., 2004; Gaines et al., 2012; Ma et al., 2015; Hammarlund et al., 2017; Qi et al., 2018; Saleh et al., 2020a). Under normal conditions, carcasses would be scavenged by other animals, decayed by bacteria, or degraded by autolytic enzymes within the carcass tissues. However, in the Chengjiang biota, these processes were relatively limited, thereby allowing the preservation and replication of soft tissues that would normally not be found in the fossils. As a result, tissues are preserved as carbonaceous films, often associated with a coating of authigenic pyrite (Ma et al., 2015; Saleh et al., 2020a). The pyrite was later weathered to form iron oxide, resulting in the characteristic rusty color of these fossils (Gabbott et al., 2004). Although the decay process was slowed down in the Chengjiang Biota, Chengjiang fossils have still undergone some anatomical decay, following death, early on during the fossilization process, and the extent of the loss of morphological characters for each group is often difficult to assess (Parry et al., 2018; Purnell et al., 2018).

Recently, taphonomic investigations based on tissue-occurrence data provided a novel tool to constrain preservation variance (Saleh et al., 2020b; Saleh et al., 2021a; Saleh et al., 2021b; Whitaker et al., 2022). This study compares the preservation of 11 major animal groups (*e.g.*, euarthropods, priapulids, brachiopods) within the Chengjiang biota, aiming to answer the following questions: What are the preservational differences between animal groups at the Chengjiang biota? What are the potential causes for these variations (from biostratinomy, to fossil collection and identification)?

When examining fossils, it is essential to understand the role that preservation plays in the presence and absence of anatomical characters. Evolutionary and ecological interpretations of fossil anatomy require a foundational basis of taphonomic context to be robust. Analyzing these questions will help in understanding how environmental conditions (*e.g.*, flow dynamics), modes of life (*e.g.*, endobenthic, epibenthic, nektonic), and biochemical compositions of tissues (*i.e.*, polysaccharides, chitin) influenced the preservation pattern of organisms during biostratinomy processes, decay, and diagenesis. It will also explore how researchers’ ability to identify and describe new taxa (Whitaker & Kimmig, 2020) might affect our understanding of preservation quality. This will help frame morphological and ecological innovations in an appropriate preservation context for the earliest, most diverse eumetazoan fossil assemblage of the Cambrian Radiation.

## Materials & Methods

The eumetazoans in the Chengjiang biota are herein divided into 11 major groups: Annelida, Euarthropoda, Brachiopoda, Chordata, Cnidaria, Ctenophora, Hyolitha, Lobopodia, Priapulida, Radiodonta, and Vetulicolia. These groups represent, for the most part, well agreed upon animal phyla. Though no classification scheme is ever without detractors and controversy (*e.g.*, Daley & Antcliffe, 2019). These divisions represent well the state of play in the field of Cambrian palaeontology. Hyoliths are considered as a separate group from Brachiopoda because the phylogenetic position of Hyolitha is debated (Liu et al., 2020a; Liu et al., 2020b). Euarthropoda, Lobopodia, Chordata, and Priapulida were chosen because they are well represented groups in terms of diversity and abundance and to allow direct comparisons with their respective data from modern decay experiments later on in the discussion. Radiodonts were also taken separately from the rest of the Euarthropoda because their mode of life as active swimmers (nektonic/nektobenthic) contrasts with most other euarthropods present at the site, which are typically epibenthic taxa (Zhao et al., 2013; Yang et al., 2021). Cnidaria and Ctenophora were included because they are more abundant in the Chengjiang biota than in other Cambrian sites with exceptional preservation such as the Walcott Quarry from the Burgess Shale of Canada (Saleh et al., 2021a). Annelida is also considered because they are so important at other Cambrian sites (*e.g.*,  Parry, Vinther & Edgecombe, 2015) despite being one of the rarest animal groups in the Chengjiang biota (Hou et al., 2017). All these groups comprise more than three genera and at least ten specimens at Chengjiang attesting for their preservational reproducibility. Chaetognatha and Hemichordata were not included in the analysis because they are monogeneric in the Chengjiang biota, with only one or two specimens of each discovered (Hou et al., 2017).

In order to investigate the preservation of different animals groups, biological tissues are divided into five categories based on their resistance to the decay process in general: A, biominerals, such as the shell of brachiopods; B, sclerotized cuticles, such as the headshield of radiodonts; C, non-sclerotized cuticles (a flexible protective covering outside the epidermis, composed of chitin, proteins and lipids), such as priapulid body walls; D, epidermis that is in direct contact with seawater, such as the body walls of chordates; E, internal organs such as digestive and nervous systems. These raw preservation data were originally published in Saleh et al. (2020b) (https://data.mendeley.com/datasets/4fhyt4fbyd/1) and are mainly based on fossils housed at Yunnan University (Hou et al., 2017) and collected from the North East of Fuxian Lake and West of Dianchi Lake in the Yunnan Province (*i.e.*, 13 sites in total: Dapotou, Ercaicun, Erjie, Fengkoushao, Hongjiachong, Jianshan, Laogaoshan, Ma’anshan, Mafang, Maotianshan, Meishucun, Shankoucun, and Xiaolantian). The average number of tissues per taxon for each group was calculated. This can be used as an index to assess the preservation of groups within the Chengjiang biota. However, its value could be impacted by the anatomical heterogeneities between groups. For instance, most living euarthropods have at least either a biomineralized or sclerotized exoskeleton, non-sclerotized cuticular parts, and internal organs, resulting in a high value (three tissue types per genus during the life of the organism). Early chordates, by contrast, have epidermis exposed to seawater in addition to internal organs (two tissue types per genus during the life of the organism). Therefore, perfectly preserved euarthropods would score higher in this index than a perfectly preserved chordate.

To constrain the effect of anatomical/taxonomic heterogeneities within the number of tissues per genus index, further analyses focus on the proportion of preserved internal organs [P(E)], in a similar fashion to what have been previously done (Saleh et al., 2020b; Saleh et al., 2021a; Whitaker et al., 2022). This has been made because all defined animal groups (except cnidarians and ctenophores that lack a mesoderm (Brusca, Moore & Shuster, 2016) and were removed from further analyses) have internal systems (while this is not necessarily true for biomineralized, sclerotized, or cuticularized parts). In this sense, P(E) and its associated 95% confidence intervals were plotted to constrain group-specific preservation in the Chengjiang biota. These 95% confidence intervals were calculated using a Bayesian approach with a null prior to account for the small sample sizes of some groups. Animal groups with a high number of preserved internal organs are considered to be better preserved in general than those with a lower proportion of fossilized internal organs. The proportion of internal organs preserved were then compared to the generic diversity and the abundance data for each group as taken from Hou et al. (2017). We acknowledge that there might be some contrast between the fossils included in this paper when compared with collections at other institutions, or even that some contrasts might exist between different localities within the Chengjiang biota. However, here we try to investigate the broad picture by comparing animal groups to each-other without focusing on the environmental disparities among outcrops. For further details regarding data collection for probabilistic investigations, methodological reasoning, and limitations, see Saleh et al. (2021a). A Spearman test was then calculated using PAST (Hammer, Harper & Ryan, 2001) in order to investigate whether a correlation exists between diversity, abundance, and the proportion of internal organ preservation (Supplementary Material).

## Results

**Figure 1:**
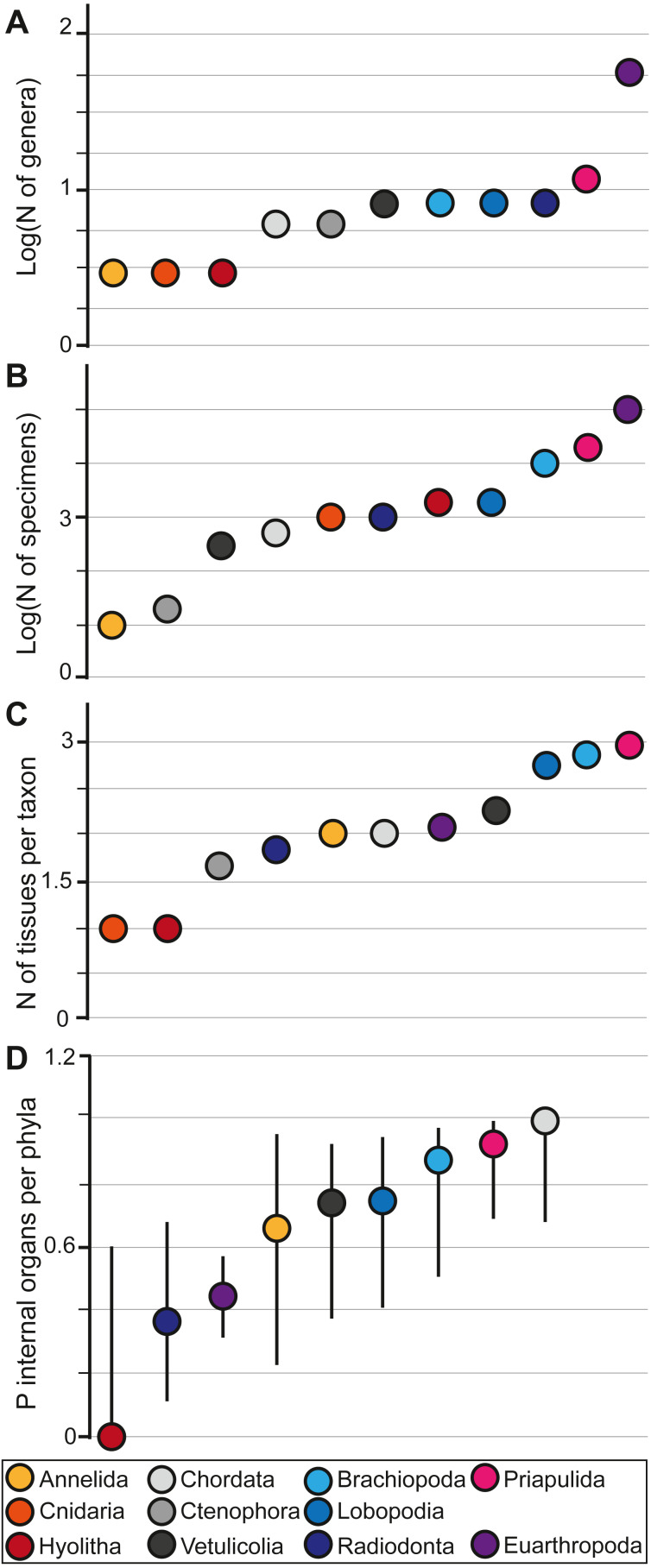
Plots showing the different indices used. The generic diversity of each group (A), the number of specimens belonging to each group (B), the number of tissues per group (C), and the proportion of internal organs within each group and its 95% confidence interval (D). Note that Cnidaria and Ctenophora are absent from D because they do not have internal organs in life. The transition between radiodonts and priapulids in D represents a transition from groups that are mainly nektonic/nektobenthic to those that are dominantly endobenthic. The logarithmic scale is used to better visualize differences between animals, as the low numbers of some groups tend to be confounded with zero when compared to the large abundances of some animals.

In the Chengjiang biota, Euarthropoda shows the highest generic diversity (*N* = 56), substantially greater than any of the other groupings and comparable in generic diversity to all the other taxa in the study combined (*N* = 65). Hyolitha (*N* = 3), Cnidaria (*N* = 3), and Annelida (*N* = 3) had the lowest generic diversity (Fig. 1A). Eurthropoda has also the highest number of individual specimens (N∼100,000) exceeding all other specimens combined by a factor of three. Whereas Annelida is represented by the lowest number of specimens, with only ten specimens in the collections of Yunnan University (Fig. 1B). Moreover, different animal groups in the Chengjiang biota do not preserve an equal number of tissues, and have contrasting proportions of internal organs. The average number of tissues per taxon increases from the lowest possible score for the Cnidaria (mean N *mN* = 1) and the Hyolitha (*mN* = 1) through to the Priapulida (*mN* = 3). All other taxa are intermediary to these with Ctenophora (*mN* = 1.66), Radiodonta (*mN* = 1.87), Annelida (*mN* = 2), Chordata (*mN* = 2), Euarthropoda (*mN* = 2.07), Vetulicolia (*mN* = 2.28), Lobopodia (*mN* = 2.75), and Brachiopoda (*mN* = 2.87) and a global average for the whole of the Chengjiang at *mN* = 2.185 (Fig. 1C). The proportion of internal organs increases from being entirely absent in Hyolitha (*P* = 0), Radiodonta (*P* = 0.37), Euarthropoda (*P* = 0.44), Annelida (*P* = 0.66), Vetulicolia (*P* = 0.71), Lobopodia (*P* = 0.75), Brachiopoda (*P* = 0.87), Priapulida (*P* = 0.91), through to being present in all genera of Chordata in at least some specimens (*P* = 1) (Fig. 1D). The proportion of internal organ preservation was found not to be correlated with generic diversity or specimen abundance (*i.e.*, Spearman test *p*-value = 0.6294 and 0.9298 respectively; Supplementary Material).

## Discussion

### Abundance vs preservation data

Internal organ preservation (Fig. 1D) is not correlated to diversity (Fig. 1A) and abundances (Fig. 1B; see Spearman test for correlation in the Supplementary Material). For example, Euarthropods have the highest number of genera as well as the highest specimen abundance (Figs. 1A and 1B). However, they do not show the highest number of tissues per taxon (Fig. 1C) and they preserve one of the smallest proportions of internal organs (Fig. 1D). Chordates, on the other hand, are not the most diverse or abundant groups in Chengjiang (Figs. 1A and 1B). However, they preserve the highest proportions of internal organs (Fig. 1D). Thus, there is a certain group-selective preservation filter at place in Chengjiang, and this selective filter is not a matter of abundances as the most diverse and abundant taxa are not the best preserved in terms of internal organs (*i.e.*, it is not simply a matter of luck aided by the large numbers of abundant and highly diverse taxa.). As a result, there must have been an interplay of both taphonomic and ecological conditions that dictated what and how animals got preserved in the Chengjiang biota.

### Biostratinomy impact on fossil preservation

The internal organ preservation sequence between radiodonts (navy) and priapulids (pink) (Fig. 1D) reflects a transition from nektonic to endobenthic taxa. In the Walcott Quarry, it has been previously suggested that endobenthic taxa are easier to capture by sedimentary flows and are often buried alive, showing little evidence for degradation when compared to actively swimming taxa (Saleh et al., 2021a). Actively swimming taxa could escape to some extent transported sediments (Saleh et al., 2021a). In this sense, what is mainly preserved for these groups are carcasses that were already decaying on the seafloor and were passively transported by flows resulting in a lower degree of preservation than endobenthic taxa (Saleh et al., 2021a). Considering that fossils in the Chengjiang biota were also transported by sedimentary flows (Saleh et al., 2022), it is possible to suggest that groups containing endobenthic taxa (*e.g.*, Brachiopoda; Priapulida) are the easiest to capture and are the best preserved (right side of the internal organ sequence; Fig. 1D). Radiodonta and Euarthropoda (left side of the internal organ sequence; Fig. 1D) contain many actively swimming genera that can escape the flow resulting in a low preservation fidelity (Fig. 1D). In this sense, the internal organ sequence “Radiodonta (nektonic/nektobenthic)—Euarthropoda (nektonic/nektobenthic/epibenthic)—Annelida (nektobenthic/epibenthic)—Lobopodia (epibenthic)—Brachiopoda (epibenthic/ endobenthic)—Priapulida (mainly endobenthic)” (Fig. 1D) represents a gradual preservational transition reflecting the interaction between sedimentary flows and the modes of life of organisms. This gradual transition could explain why no significant differences are observed when comparing consecutive animal groups one by one to each other, and significant differences are mainly observed between the extremes of this sequence (priapulids and radiodonts; Fig. 1D). Early in the Cambrian, the functional and ecological diversifications have not yet been unfolded, and each animal group was closely tied to a limited number of ecological roles (Bambach, Bush & Erwin, 2007). As a result, the phylogenetic signal in preservation variation is similar to that of the mode of life/ecological signal.

Hyolitha and Chordata are outliers to this sequence (Fig. 1D). For instance, hyoliths are epibenthic (maybe shallow endobenthic) but they are on the extreme left side of the internal organs sequence (Fig. 1D). Chordates are nektobenthic/nektonic but they plot on the extreme right side of the internal organs sequence (Fig. 1D). This pattern may therefore have been induced by favorable chemical conditions for chordate preservation at Chengjiang (Saleh et al., 2021a). These conditions can vary from those inhibiting the decay to those favoring the replication of chordate internal tissues in authigenic minerals or both (Parry et al., 2018; Purnell et al., 2018). On the contrary, it appears that hyoliths internal organs decayed faster than the rest, and were not preferentially stabilized through mineral precipitations at this site (Fig. 1D). This indicates that taphonomic processes acted differentially on the soft tissues across the major animal groups. This resulted in wildly variable preservation quality of functionally similar and homologically evolved bilaterian organs which nevertheless can be constructed of different materials depending on the animal group.

Some differences might exist between different outcrops at Chengjiang, likely reflecting the various depositional settings represented within this Lagerstätte (Saleh et al., 2022). However, considering that most flows transported organisms from their habitat to environments favorable for their preservation (Saleh et al., 2022), the impact of any of these flows will remain more pronounced on the endobenthic community in comparison to epibenthic, nektobenthic, and nektonic taxa (regardless of the precise sedimentary sub-environment investigated and the proportion of deposits associated with a particular flow in a given locality). The only exception within the sedimentary environment of the Chengjiang biota is hemipelagic deposits where no flows are recorded (Saleh et al., 2022). However, this setting is not accounted for herein, because it yielded a low diversity of fossils mainly consisting of sponges (Qi et al., 2018). Sponges are not included in this study, because they are not formed of the same types of tissues, and body plans, as eumetazoans. In this context, while specific outcrop data is unlikely to alter the main modes of life hypothesis of this paper, further evaluation must be done in the future. For example, chordates that are outliers to the mode of life hypothesis presented earlier differ in their preservation between outcrops (Yang et al., 2021). Outcrop comparisons will likely lead to the refinement of the results presented herein and resolve some of these subtle preservational variations.

### Comparisons with decay experiments

**Figure 2:**
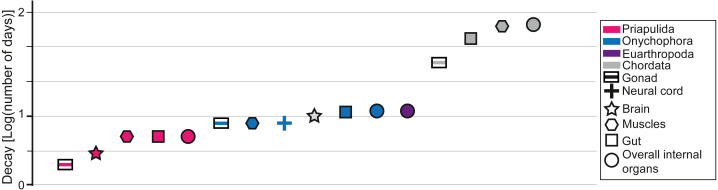
Data from decay experiments. Time of complete degradation of numerous internal organs in Priapulida (Sansom, 2016), Onychophora (Murdock et al., 2014), and Chordata (Sansom, Gabbott & Purnell, 2010) as observed in decay experiments. Note that for Euarthropoda (Butler et al., 2015) there is no differentiation between internal organs in the literature as only the time of disappearance of all tissues is considered.

Decay experiments on modern taxa are a powerful tool that provides an alternative view of the patterns observed in the fossil record (Briggs & Kear, 1993a; Briggs & Kear, 1993b; Briggs & Kear, 1994; Sansom, Gabbott & Purnell, 2010; Sansom, Gabbott & Purnell, 2011; Sansom, 2014; Sansom, 2016; Murdock et al., 2014; Butler et al., 2015; Klompmaker, Portell & Frick, 2017; Hancy & Antcliffe, 2020). Decay experiments have been performed on many experimental models, such as euarthropods (Briggs & Kear, 1994; Butler et al., 2015; Klompmaker, Portell & Frick, 2017), onychophorans (close to lobopodians; Murdock et al., 2014), chordates (Briggs & Kear, 1993a; Briggs & Kear, 1993b; Sansom, Gabbott & Purnell, 2010; Sansom, Gabbott & Purnell, 2011), priapulids (Sansom, 2016), and cnidarians (Hancy & Antcliffe, 2020), providing a baseline for most of the major groups found in the fossil record of the Chengjiang biota. Most decay experiments were performed under limited environmental conditions (*e.g.*, in the absence of sediments). Some decay experiments were performed with the presence of a substrate and indicated that animals could eventually get preferentially preserved in the presence of clays, in particular kaolinite (Wilson & Butterfield, 2014; Naimark et al., 2016a; Naimark et al., 2016b). Despite the limitations of decay experiments they can still be used to confirm a certain finding or to highlight where a gap in understanding exists (Purnell et al., 2018). For instance, it was experimentally shown that nervous tissues in numerous groups decay particularly fast in laboratory conditions (Fig. 2). However, numerous brains were discovered in many taxa from the Chengjiang biota (*e.g.*, Ma et al., 2012; Ma et al., 2015). It was subsequently demonstrated that neural tissues in Chengjiang animals were replicated in authigenic minerals very rapidly following the death of the organism allowing for their preservation in the rock record to the exclusion of other tissues (Saleh et al., 2020a). This taphonomic model shows that information from decay experiments and the pattern of fossil preservation are complementary evidence for which explanatory processes can be discovered (Saleh et al., 2020a). As such, by investigating whether the increase in the proportion of internal organ preservation (Fig. 1D) reflects an increase in decay resistance (Fig. 2), it becomes possible to test some of the hypotheses that emerged in the previous sections. It is shown in decay experiments that the degradation of chordate internal organs is slower than in other groups such as Priapulida, Onychophora, and Euarthropoda (Fig. 2). This has been clearly seen in the fossil record of the Chengjiang biota (Fig. 1D). However, in decay experiments, it is observed that Priapulida decay faster than Onychophora, and Euarthropoda respectively (Fig. 2). An opposite pattern has been observed in the Chengjiang biota for these animal groups with Euarthropoda being faster to decay in comparison to Priapulida (Fig. 1D). Thus, there must have been other parameters influencing the sequence of decay at Chengjiang. The mode of life hypothesis suggested in this study can explain the contrast between the results of decay experiments and the fossil record. This hypothesis suggests that endobenthic groups such as Priapulida cannot escape sedimentary flows and are often buried alive (facing a very short decay time). Moreover, if dead prior to their transport, endobenthic taxa (*e.g.*, Cambrian priapulids) are automatically buried at deeper levels within the sediment, slowing down their decay (Pratt & Kimmig, 2019). Epibenthic, nektobenthic, and nektonic taxa are able to escape, to some extent, the flow and only exposed carcasses of these animals are preserved (very long decay time). The interaction between modes of life and preservation is not limited to the Chengjiang biota and other Burgess Shale-type deposits. For example, it was long assumed that the rarity of ten-armed Decabrachia (squid and cuttlefish) in comparison to the relatively abundant phosphatized eight-armed Vampyropoda (octopuses and vampire squid) among cephalopod molluscs in phosphatic Mesozoic Lagerstätten is ecological. However, it was recently demonstrated that it is largely caused by differences in their chemical composition and buoyancy mechanism during life (Clements et al., 2017). Subsequent studies showed that Decabrachia and Vampyropoda overlapped in their habitats meaning that differences cannot be attributable to ecology *sensu stricto* (Košák et al., 2021). The first soft-tissue squid was also found in non-phosphatic sites further corroborating the interaction between preservation and the chemistry of the physiology (Mironenko et al., 2021).

Even though the slow decay of chordate tissues and the interaction between modes of life and sedimentary flows explain the patterns observed in the Chengjiang biota, it is important to acknowledge that there might be other explanations for this pattern. For instance, changing the conditions in decay experiments can often lead to contrasting results. It has been recently shown that some cnidarian tissues decay faster under anoxic conditions than in the presence of oxygen (Hancy & Antcliffe, 2020). Euarthropod tissues decay substantially slower under antimicrobial reducing conditions than in the presence of oxygen (Butler et al., 2015). In the future, it is essential to decay animal groups under various environmental conditions in order to build numerous experimental decay sequences that can be then compared to the patterns observed in the fossil record.

### Other factors leading to preservation variation

**Figure 3:**
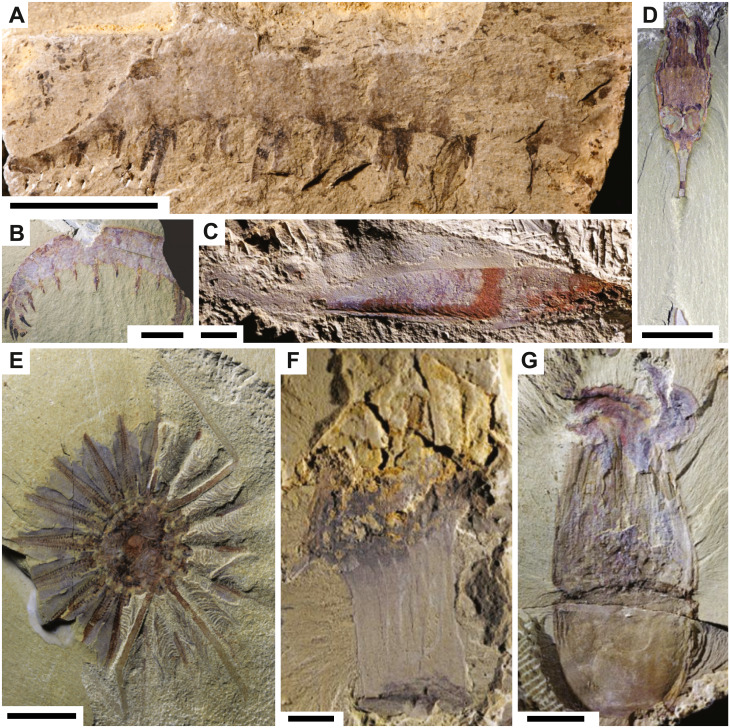
Fossils from the Chengjiang biota. Radiodonts: (A) *Anomalocaris* cf. *canadensis* JS-1880 (Wu et al., 2021), (B) *Laminacaris chimera* CJHMD-00003 (Guo et al., 2019). Chordate: (C) *Myllokunmingia fengjiaoa* RCCBYU-10200a (Hou et al., 2017). Ctenophores: (D) *Dinomischus venustus* YKLP-13412b (Zhao et al., 2019), (E) *Daihua sanqiong* YKLP-13401a (Zhao et al., 2019). Cnidarians: (F) *Archisaccophyllia kunmingensis* YKLP-10379 (Hou et al., 2017), (G) *Xianguangia sinica* YKLP-13830 (Hou et al., 2017). Scale bars: 10 mm (A, E, F, G), 20 mm (B), one mm (C), five mm (D). JS (Northwest University), CJHMD (Chengjiang Fossil Museum of the Management Committee of the Chengjiang Fossil Site World Heritage), both YKLP and RCCBYU are at Yunnan Key Laboratory for Palaeobiology (Yunnan University).

The contrasting patterns of different groups at Chengjiang may not be resulting from differential preservation but rather from the ability of paleontologists to recognize a particular fossil. Euarthropoda and Radiodonta remains are generally easily recognizable in the rock record (Guo et al., 2019; Wu et al., 2021). For instance, in sites with exceptional fossil preservation, many radiodonts are known from their molting products, with complete carcasses being extremely rare. In the Walcott Quarry, only one complete specimen of *Anomalocaris* is known, but tens of fossil fragments can be confidently attributed to this genus based on key morphological features (Nanglu, Caron & Gaines, 2020). In the Fezouata Shale, complete carcasses of the radiodont *Aegirocassis* are similarly relatively rare (Saleh et al., 2020b), yet many fragments were easily assigned to this genus (Van Roy, Briggs & Gaines, 2015). The easy recognition of euarthropod and radiodont fossil fragments to existing or new genera (Zhu, Lerosey-Aubril & Ortega-Hernández, 2021) may account for the lower degree of preservation observed within these groups because most fossil fragments will be deprived of internal organs, resulting in data entry without the most labile parts (Figs. 3A and 3B). This does not arise for other major groups of animals. The designation of a specific fossil to the Chordata, for example, requires the preservation of very specific and labile characters. To avoid any ambiguity and uncertainty, paleontologists tend to define new species/genera based solely on almost complete specimens of chordates. Those complete specimens are often preserved with internal parts and thus, logically show a higher degree of preservation (Fig. 3C). Ctenophores and cnidarians are other examples of this dilemma (Figs. 3D–3G). Paleontologists often rely on complete specimens in order to say if a particular fossil belongs to cnidarians or ctenophores and even then, it is contentious (Daley & Antcliffe, 2019). It is almost impossible to recognize any fossil fragment belonging to these groups if it has not been described previously. Even when nearly complete specimens are discovered, it will be still challenging to assign those carcasses to either cnidarians or ctenophores because defining and recognizing characters can be ambiguous (Daley & Antcliffe, 2019; Zhao et al., 2019).

The possibility of paleontologists unconsciously and unwillingly inducing variations in a certain dataset is not limited to the ability to identify a certain fossil fragment. Preservational variations can also be induced by fossil collection, preparation, accession, and digitization (Whitaker & Kimmig, 2020). Variations can be induced following certain practices such as sampling from a preferred locality, or the background, interest, and specialization of the sampling team (Whitaker & Kimmig, 2020). However, within the context of this paper, we consider that induced preservational variations remain minimal. Sampling for the fossils that are currently housed at Yunnan University was always made by a group of international researchers covering numerous aspects of paleontological research. Future investigations can reveal different patterns in fossil collections at other institutions. Quantifying the divergence between institutional collections will be key to quantifying collection biases and understanding the impact we and other colleagues might unconsciously introduce in databases.

## Conclusions

The preservation potential of the Chengjiang biota was analyzed using occurrence data. It is shown that abundant groups are not necessarily the best preserved in terms of internal organs. The differences observed between groups can be explained by the ecology of living animals, and by processes such as transport and decay. It is worth noting that the results of decay experiments and fossil-based datasets are complimentary in resolving preservational patterns in the rock record and are mutually illuminating as we search for a deeper understanding of complex preservation processes. The differences in preservation observed between animal groups within the Chengjiang biota highlight that future taphonomic investigations should be made at an animal group level in order to properly understand the mechanisms of preservation within a certain site and between sites. Such understanding would be ungranted when taking sites as a whole, without data partitions between animal groups. However, various complications of collection and taxonomic practice might be affecting preservation signals when done at a group-level, particularly with respect to how easily diagnostic characters are recognized by researchers. For example, many euarthropod fossils from Chengjiang preserved internal organs (such as vascular systems) that are not found in other animal groups (Ma et al., 2014). Yet, the preservation of arthropods, using this probability model is shown to be worse than other animal groups never preserving these organs. This contrast could be caused by the ability of researchers to associate, among non-mineralized fossils, euarthropods fragments (that lack internal organs) to new species/genera (whereas it is almost impossible to describe a chordate fragment as a new species), often leading to an underestimation of the preservation potential of arthropods when taken as a whole.

##  Supplemental Information

10.7717/peerj.13869/supp-1 Supplemental Information 1 Raw occurrence data used to obtain the results in addition to the Spearman correlation test Occurrence data is inventoried (1 = present; 0 = absent). When the *p*-value of the Spearman test is above 0.05, it means that there is no correlation.

## Additional Information and Declarations

### Data Availability

The following information was supplied regarding data availability: Raw data is available in the Supplemental Files.
